# Label-Free, Real-Time Measurement of Metabolism of Adherent and Suspended Single Cells by In-Cell Fourier Transform Infrared Microspectroscopy

**DOI:** 10.3390/ijms221910742

**Published:** 2021-10-04

**Authors:** Tommaso Vannocci, Luca Quaroni, Antonio de Riso, Giulia Milordini, Magda Wolna, Gianfelice Cinque, Annalisa Pastore

**Affiliations:** 1 UK Dementia Research Institute at The Wohl Institute of King’s College London, London SE5 9RT, UK; tommaso.vannocci@kcl.ac.uk (T.V.); giulia.milordini@iit.it (G.M.); 2 Department of Physical Chemistry and Electrochemistry, Faculty of Chemistry, Jagiellonian University, 30-386 Kraków, Poland; 3 Evotec (UK) Ltd., Dorothy Crowfoot Hodgkin Campus, Milton Park, Abingdon OX14 4RZ, UK; antonio.de.riso.bioch@gmail.com; 4 MIRIAM beamline B22, Diamond Light Source, Harwell Campus, Didcot OX11 0DE, UK; magda.wolna@yahoo.co.uk (M.W.); gianfelice.cinque@diamond.ac.uk (G.C.)

**Keywords:** cellular metabolism, glycolysis, infrared microscopy, synchrotron infrared, cellular adhesion

## Abstract

We used infrared (IR) microscopy to monitor in real-time the metabolic turnover of individual mammalian cells in morphologically different states. By relying on the intrinsic absorption of mid-IR light by molecular components, we could discriminate the metabolism of adherent cells as compared to suspended cells. We identified major biochemical differences between the two cellular states, whereby only adherent cells appeared to rely heavily on glycolytic turnover and lactic fermentation. We also report spectroscopic variations that appear as spectral oscillations in the IR domain, observed only when using synchrotron infrared radiation. We propose that this effect could be used as a reporter of the cellular conditions. Our results are instrumental in establishing IR microscopy as a label-free method for real-time metabolic studies of individual cells in different morphological states, and in more complex cellular ensembles.

## 1. Introduction

One facet of cellular metabolism that is receiving increasing attention is the relationship between cellular morphology, adhesion, and metabolic state of the cell. It is nowadays recognized that metabolic changes accompany the morphological evolution of a cell and its interaction with the extracellular microenvironment [1,2]. Recent advances have related the metastatic capacity of cancer cells to changes in their balance between glycolysis and oxidative phosphorylation that correlate with adhesion [3,4]. Addressing the interplay of cellular metabolism and morphology calls for analytical methods with the capability to quantitatively monitor metabolite concentration in time and space across living cells at single-cell resolution. Most conventional methods for metabolic investigations require the use of bulk samples, which implies, by definition, sample averaging and prevents the possibility to associate metabolic activity to a specific cellular entity. Techniques that allow the imaging of cellular metabolism often require some form of labelling or sample enrichment, which restrict the investigation to pre-selected targets. Mass spectrometry provides the highest sensitivity and specificity, but the application to live cell sampling, using ambient ionization methods, requires at last partial cytoplasmic extraction, preventing recording of time-resolved events [5]. An optimal method should be quantitative, non-invasive, require minimal or no sample pre-treatment, and be applicable to cell culture models and living organisms. It should afford molecular specificity and the capability to simultaneously detect multiple molecular species, ideally without a priori target selection. Finally, it should also provide the possibility to monitor the activity of both single cells and cellular ensembles in real time. Most of the desired features are provided by mid-infrared (IR) absorption spectromicroscopy [6].

Absorption spectroscopy in the mid-IR spectral region (wavelength approx. 2.5–25 µm; from now on, simply IR spectroscopy) measures vibrational transitions in matter, providing information on composition, structure, and dynamics at the molecular level. Because of its compositional sensitivity, it is often used for analytical applications in the biological sciences. In addition, its sensitivity to molecular structure and its applicability to a range of sampling conditions have been extensively utilized for the study of biomolecules and their dynamics in bulk samples.

The combination of IR spectroscopy with microscopy has enhanced the method by providing spatial information [7,8], bringing the added value of microscale resolution in the spatial distribution of molecular composition and structure. The possibility of using synchrotron radiation sources coupled to Fourier Transform IR (FTIR) has further widened applications by increasing the spectral quality (signal-to-noise ratio) at the highest spatial resolution (diffraction-limited) optically allowed in IR microanalysis [9].

Spectromicroscopy in the mid-IR spectral region (from now on, simply called IR microscopy) was adapted to gain both qualitative and quantitative information on complex biological systems, such as tissues [10] and cells [6,11], and has recently received renewed attention as a tool for monitoring real-time metabolic activity at the level of multiple and the single eukaryotic cells [12]. IR microscopy allows the study of even complex biospecimens without the need of chemical modification such as molecular labelling, staining, or other sample alterations, thus making it a fully non-invasive untargeted technique [13]. It can be performed on both adhered [14] and suspended [15] cells, allowing the study of both biologically relevant states.

FTIR spectroscopy on macroscopic samples, while allowing the screening of extended samples with better signal-to-noise ratio and higher sensitivity than its microscopy implementation, also delivers ensemble averaged values. It is therefore unable to inform about the properties of individual cells, to relate cellular properties to spatial location and/or morphology, to inform on low abundance phenotypes, or to discriminate cellular from extracellular properties.

Here, we experimentally test the capability of IR microscopy to monitor individual cells from the same line but with widely different morphologies. We used a HEK-derived cell line [16] to assess whether it is possible to discriminate the metabolic turnover in cell types with widely different morphology, namely adherent and suspended cells. We benchmarked the applicability of IR microscopy using both synchrotron radiation and thermal sources and demonstrated that the method allows detecting the distinct metabolic activity of the two cell morphologies. This conclusion will be used in future work aiming to investigate the properties of specific cells and their environment within complex cellular assemblies, where the identity, morphology and cell cycle phase of individual cells can vary in space and time.

## 2. Results and Discussion

**Figure 1:**
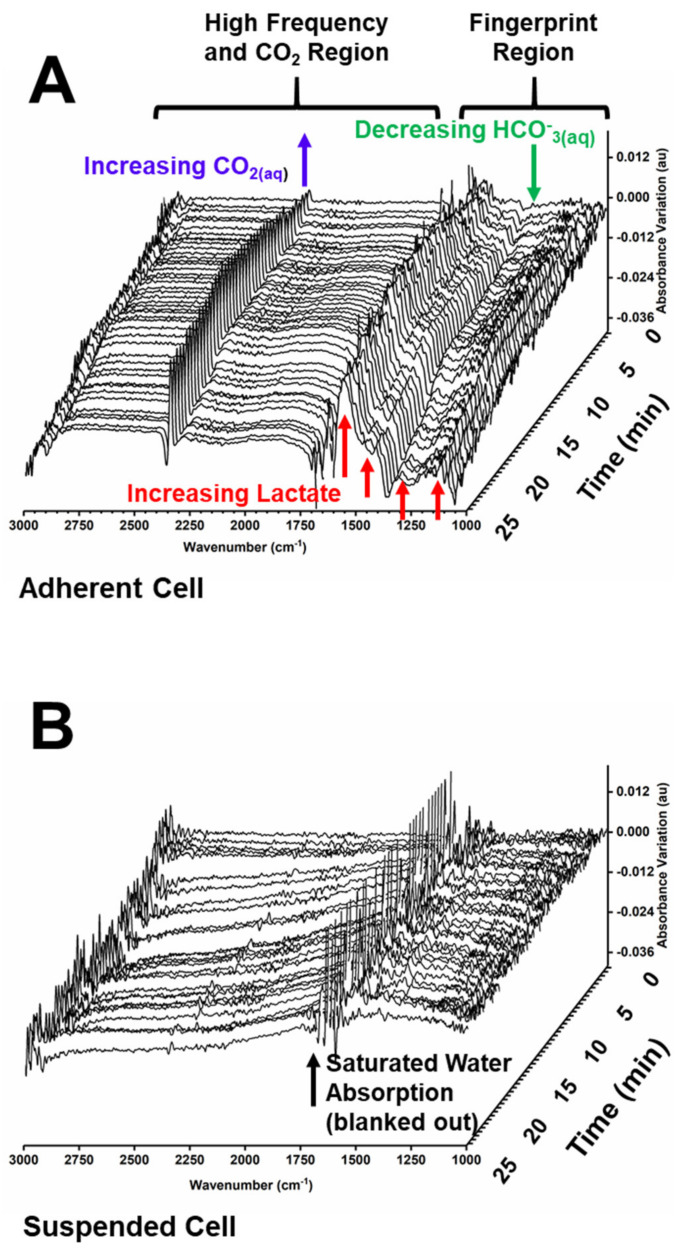
Spectral variations of single adherent and suspended cells over 25 min. (**A**). Single Adherent Cell. The brackets define the fingerprint spectral region and the high frequency region. The arrows show the increasing absorption bands from lactate (red) and CO_2(aq)_ (blue) and the decreasing absorption band from HCO_3_^−^_(aq)_ (green). (**B**). Single Suspended Cell. The arrow shows the position of the saturated absorption band from water, dominated by noise (points removed from both A and B for clarity).

We performed a systematic study of HEK-*cFXN* single-cells in both adherent and suspended conditions. Cells were enclosed in the sample holder (described in Supporting information), isolated from the atmosphere, and monitored under the IR microscope over a 25-min interval without circulating fresh medium. The evolution of their biochemical and biophysical properties was assessed by recording spectral variations in the range of 1000 cm^−1^–3050 cm^−1^ using both synchrotron radiation and a thermal source (Figure 1). The optic geometry implied that, in the case of the suspended cells, the footprint of the IR microbeam from the thermal source covered a whole single cell plus part of the surrounding medium. The suspended cells were about 10 µm thick, i.e., comparable to the spacer thickness, and were sandwiched in contact with the two optical windows, leaving no headspace. Adherent cells were stretched over the surface, with an approximately polygonal shape, and estimated thickness between 2 µm to 5 µm. Therefore, they were thinner than the 10 µm spacer used in the sample holder, leaving a corresponding headspace which was also probed by the IR beam. In the case of adherent cells, most of the cell core was probed in the measurement, except for the longer cellular extensions. Figure 1 highlights the spectral regions analysed. The fingerprint region contains absorption bands from most of the molecular components of the cells. The fine variations in band intensity in this region, if decoded, can provide detailed information on all the ongoing chemical processes within the cell and its immediate environment [17]. The higher wavenumber region mostly displays absorption bands from C–H-containing molecules, plus carbon dioxide, within the sample and the atmosphere. The interval of the absorption band from the water bending mode, between 1680 cm^−1^ and 1610 cm^−1^, was removed in the displayed spectra because of the high noise levels.

The spectral variations displayed by the adherent and suspended cells were remarkably different, with suspended cells showing small irregular variations over time, while clear changes were observed in the spectra of the adherent cells. When observed, the variations were dominated by the increase and decrease of multiple bands in the fingerprint region. By recording the cell position at the beginning and at the end of each measurement, we could associate many of the observed variations to cellular movements, and/or to changes in cell morphology, such as swelling, all of which can perturb IR spectra by changing the amount of material probed by the beam. For this reason, we restricted our analysis to the dominant spectral changes. In addition, we discarded cells that displayed major structural rearrangements or displacement over the course of the experiment to perform quantitative assessments.

The most obvious spectral difference between the two cell morphologies was the increase in absorption from aqueous carbon dioxide (CO_2(aq)_), which dominated the spectra of adherent cells, while being absent from the spectra of suspended cells (Figure 1). The ν_3_ absorption band of CO_2(aq)_, observed at 2343 cm^−1^, overlapped with the interfering absorption from the atmospheric carbon dioxide (CO_2(atm)_), which had to be removed by subtraction. Observed variations in absorbance units, in the range of 2–3 mAU over 25 min, can be converted to concentration changes using published absorption coefficients (1.5 × 10^−4^ µm^−1^ mM^−1^) [18], indicating an increase of CO_2(aq)_ concentration in the 20 mM range in the proximity of single adherent cells.

**Figure 2:**
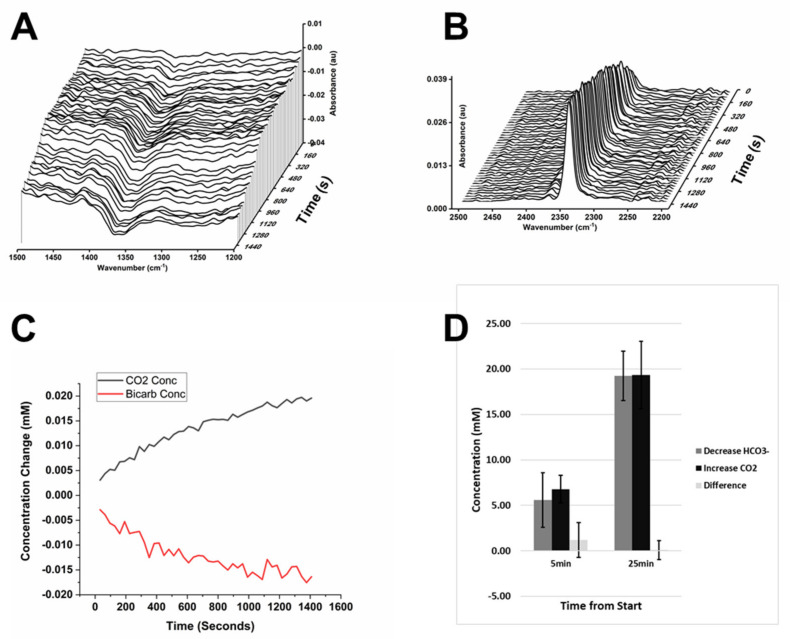
Comparison of formation of CO_2(aq)_ and consumption of HCO_3_^−^_(aq)_ in single cell measurements. (**A**). Decrease of the HCO_3_^−^_(aq)_ absorption band at 1361 cm^−1^. (**B**). Increase of the CO_2(aq)_ absorption band at 2343 cm^−1^. (**C**). Changes in the concentration of CO_2(aq)_ and of HCO_3_^−^_(aq)_ corresponding to the absorption changes in A and B. (**D**). Comparison of CO_2(aq)_ formation and HCO_3_^-^_(aq)_ consumption in individual adherent HEK-*cFXN* cells with normal expression of frataxin and the difference between CO_2(aq)_ formation and HCO_3_^−^_(aq)_ consumption calculated for each cell. Means from three cells (error bars show ± σ interval).

In parallel to the increase of CO_2(aq)_ absorption, a decrease in an absorption band at 1361 cm^−1^ was observed in adherent cells. The band was attributed to a diminution in the concentration of aqueous bicarbonate (HCO_3_^−^_(aq)_). The HCO_3_^−^_(aq)_ absorption falls in the much more crowded fingerprint region than the absorption band of CO_2(aq)_. Nonetheless, variations were generally larger than any other surrounding changes, allowing an estimate of the absorption change. Furthermore, when observed, the variations were always negative and unaffected by cellular movements, most likely indicating that they correspond to a decrease of the extracellular bicarbonate concentration. Using an absorption coefficient of 0.4 × 10^−4^ µm^−1^ mM^−1^ [18], we estimated variations in concentration in the 20 mM range over 25 min, comparable to the variation of CO_2(aq)_. (Figure 2) The correspondence and magnitude of the variations exclude major contributions from extracellular reactions or non-physiological processes, such as the dissolution of atmospheric CO_2_.

Concerted variations in the concentration of HCO_3_^−^_(aq)_ and CO_2(aq)_ indicated that the formation of CO_2_ was caused by the protonation of bicarbonate, corresponding to a pH decrease. The protonation of bicarbonate forms carbonic acid, which rapidly dissociates into CO_2(aq)_ and water [18]. The volume probed by the infrared beam includes the cytoplasmic space and the extracellular space, implying that measured spectroscopic changes describe the average response over the two volumes. In the assumption that the buffering capacity is dominated by bicarbonate, the local pH value following acidification could be estimated from the change in concentration of CO_2(aq)_ and HCO_3_^−^_(aq)_ [17], corresponding to final pH values of approximately 5.9 to 6.1.

Extracellular acidification is the standard reporter of a cellular metabolism dominated by glycolysis and pyruvate fermentation. Fermentation of pyruvate generates lactate as the final product of the catabolism of glucose, which is then secreted into the extracellular environment. Lactate secretion is usually the direct cause of extracellular acidification via the action of the monocarboxylate transporter, which transfers one proton per lactate molecule to the extracellular environment. Accordingly, the measured acidification in these experiments was comparable to the values we had previously reported from IR microscopy of A549 cell cultures [17].

**Figure 3:**
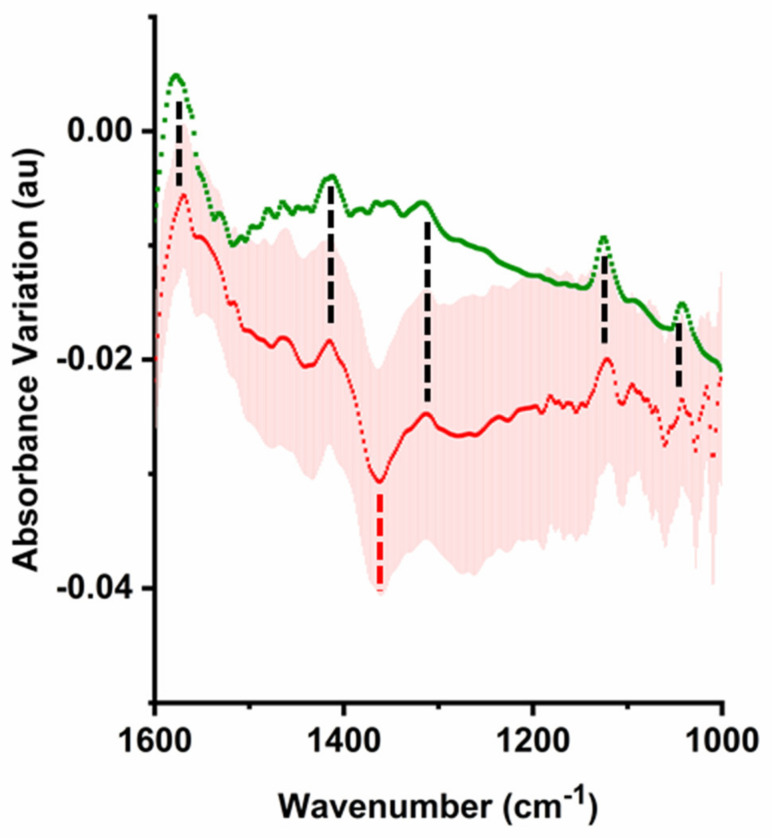
Detection of Lactate Formation. Comparison of the differential absorbance in the fingerprint region after 25 min averaged over 3 cells (the red line shows the mean of the spectra, while the shaded area shows values of ±σ at each wavenumber), with the absolute absorbance of 10 mM sodium lactate (green line). The dashed black lines highlight increasing lactate absorption. The dashed red line highlights decreasing bicarbonate absorption.

To confirm that acidification prevalently arose from glycolytic metabolism, we analyzed the spectra for the presence of lactate. The fingerprint region of adherent cells displayed a wealth of small absorption changes, arising from multiple cellular processes. The analysis of the pattern of these variations in the spectra of monolayers of A549 cells had previously revealed the formation and consumption of small molecules, including lactate [17]. A similarly detailed analysis was not feasible on the single cell samples analyzed in the present work because of the lower signal-to-noise ratio obtained in single cell spectra. Nonetheless, bands attributable to lactate became detectable in the spectra of single adherent cells after measuring for several minutes, although with low intensity. Comparison with the spectrum of sodium lactate, confirmed the identification of multiple lactate bands (1573 cm^−1^, 1413 cm^−1^, 1314 cm^−1^, 1125 cm^−1^, 1043 cm^−1^). The lactate bands increased over time, in parallel with the decrease of the band of bicarbonate (1360 cm^−1^) and the increase of the band of CO_2(aq)_ (2343 cm^−1^) (Figure 1). To corroborate the identification, we improved the signal-to-noise ratio by averaging three spectra of single adherent cells (Figure 3). A positive variation of the bands was observed in multiple cells. The average local lactate variation over 25 min was estimated to be in the millimolar range by comparison with the spectra of sodium lactate. This was comparable, although somewhat lower, to the observed formation of CO_2(aq)_ and consumption of HCO_3_^−^_(aq)_, thus reinforcing the correlation between acidification and lactic fermentation.

In addition to being generated by acidification of the medium, production of detectable CO_2(aq)_ was expected from turnover within the citric acid cycle and from other metabolic pathways [19]. In our experiments, the increase of CO_2(aq)_ in adherent cells was closely matched to the consumption of HCO_3_^−^_(aq)_, indicating a negligible contribution from metabolic CO_2(aq)_ production. Surprisingly, no CO_2(aq)_ was detected in measurements on suspended cells, even during the first few minutes of the measurements.

The measurements reported in the previous sections were repeated using synchrotron radiation IR. The synchrotron infrared beam can be focused to a diffraction-limited light spot via the same microscope optics used for the thermal source. The smaller dimensions of the synchrotron IR spot allowed selective probing of suspended cells with minimal contribution from the surrounding medium. Measurements with the synchrotron source confirmed the difference in CO_2(aq)_ and HCO_3_^−^_(aq)_ turnover already reported between adherent and suspended cells. However, they also revealed an unexpected optical effect not observed with the thermal source.

**Figure 4:**
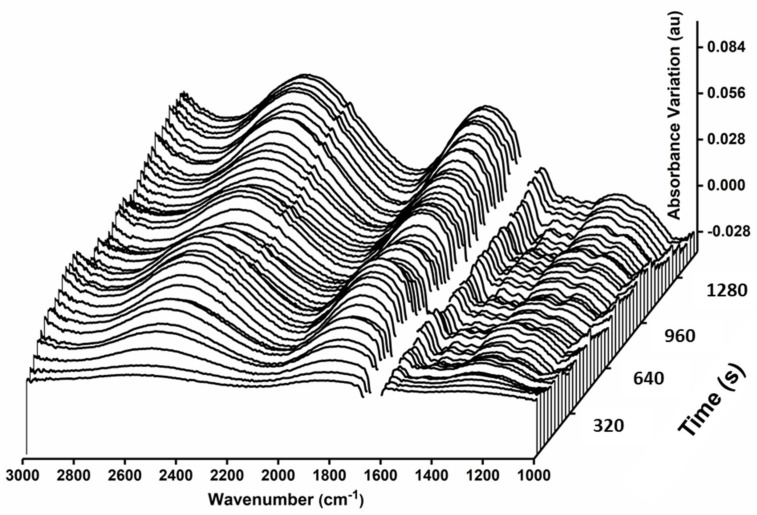
Effect of Synchrotron radiation on Single Cells. Time course of the spectrum of a single suspended cell when measuring with synchrotron radiation. Spectra measured at 32 s intervals over a total of 25 min. The interval between 1600 cm^−1^ and 1700 cm^−1^ shows saturation of water absorption and has been removed for clarity.

A distinguishing feature of all spectra recorded with synchrotron light, from both adherent and suspended cells, was the contribution from baseline variations that evolved over time (Figure 4). The variations appeared as broad spectral fluctuations, with a variable apparent period of the order of several hundred wavenumbers (ca. 700–1000 cm^−1^), and extending throughout the mid-IR spectral region, including the 1800–2500 cm^−1^ interval where biological matter typically shows few or no IR-active modes. In all cases, they developed progressively during the measurement, as the microbeam was maintained in the same sample position. However, they were not seen if the beam position was translated during the experiment. They were exclusively linked to cell presence and were not observed when measuring the medium in the sample holder, thus suggesting an interplay between the properties of the cellular matter and specific characteristics of the IR source. We did not observe oscillations with such structure when measuring dried cells. The latter observation, together with the observed time evolution, indicated that they arise from the properties of intact cells following protracted exposure to the synchrotron beam.

When compared to radiation from a thermal source, synchrotron radiation is mainly characterized by a uniquely broadband spectrum, including the whole IR region, a greater degree of spatial and temporal coherence, and a pulsed time structure. Diamond beamline B22, used in these experiments, extracts radiation over the 10,000 to 5 cm^−1^ spectral range, which is limited by the FTIR beam splitter and filter used in our specific experiment to allow only infrared radiation to reach the sample. The fluctuations could be interpreted as interference fringes from variations in the real part of the refractive index of the cell during the measurement, perhaps enhanced by the coherence of the synchrotron emission [20]. Our first hypothesis is that such spectral oscillations could be related to dynamic changes of the refractive index and geometrical properties of the multiple layers of material crossed by the microbeam. Optical variations involving the headspace between cell and optical windows (for adherent cells) and the properties of subcellular structures, including the nucleus, membrane stacks, coacervates and organelles, could all contribute to these effects. The observation of the optical effect with both adherent and suspended cells, which have widely different geometry, with no headspace present for the latter, proved that it is related to cellular presence under the beam, and not to the geometry of the headspace.

An alternative, or additional, interpretation is that the observed fluctuations could arise from changes in collective vibrations within the cell, such as the absorption of hydrogen bonded networks of water molecules [21,22], and/or from coupling to vibrations of the cellular skeleton or of the membranes. Both interpretations require the cells to be intact, highlighting the potential usefulness of these oscillations, which become visible in IR microscopy by the use of a diffraction limited, pulsed, and coherent source, as a physical marker of cellular viability.

**Figure 5:**
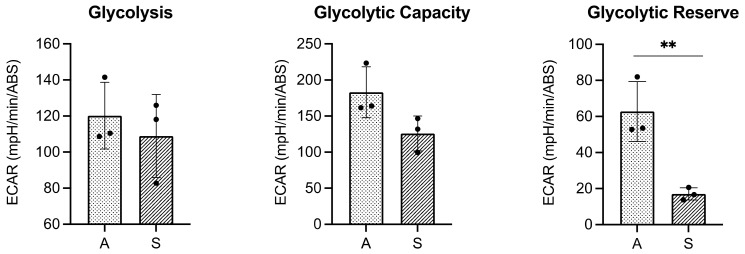
Glycolytic stress assay to validate the FTIR results. The assay was performed by measuring ECAR variations of adherent (A) and suspended (S) cells during time. Glycolysis, glycolytic capacity, and glycolytic reserve were obtained by sequentially treating the samples with glucose and oligomycin. Data are means ± SD of *n* = 3 independent experiments. Each experiment is expressed as the mean of three technical replicates, each replicate being composed by 12 biological samples (1 × 10^5^ cells per sample). The recorded signals were normalized against total protein content (expressed as an absorbance value, ABS). A positive variation corresponds to a decrease of pH. Data were analysed by t-test (** *p* ≤ 0.01).

Finally, we validated the results by assessing possible differences in glycolysis, the glycolytic capacity, and the glycolytic reserve between adherent and suspension HEK-*cFXN* cells by measuring the extracellular acidification rate (ECAR) by a Seahorse XF96 extracellular flux analyser (Agilent). The experiment, termed glycolytic stress assay, relies on monitoring the variation of extracellular pH during the sequential addition of glucose, oligomycin (ATP synthase inhibitor) and 2-deoxy-glucose (2-DG, a glucose analogue) compounds to the medium of glucose-starved cells. Glycolysis under basal conditions and glycolytic capacity of suspension cells compared to adherent cells were unaffected while we detected a strong, statistically significant ( *p* ≤ 0.01) 73% reduction in glycolytic reserve of suspended as compared to adherent cells (Figure 5). The glycolytic reserve, calculated as the difference in ECAR between the glycolytic capacity and basal glycolysis, is a measure of the cell’s ability to compensate through glycolysis when there is an increase in energy demand. Our results indicated a reduced ability of suspended cells to compensate for high energy demand compared to adherent cells, independently confirming the FTIR results.

## 3. Conclusions

In this work, we used IR microscopy to monitor the metabolic activity of suspended versus adhered living HEK-derived cells by relying on the intrinsic IR light absorption from multiple molecular species in the sample. By monitoring variations in the concentration of CO_2_, HCO_3_^-^, and lactate, we quantified the metabolic activity at the single cell level, revealing large metabolic differences between adherent and suspended cells. Whereas adherent cells displayed the hallmarks of a metabolism relying on glycolysis followed by lactic fermentation, suspended cells did not display any metabolic activity within the detection limits of IR microscopy. We also identified a spectral effect that appears to arise from the interplay of synchrotron infrared radiation and the cells and manifests itself as oscillations in the spectral domain that slowly develop over time. The effect deserves further investigation to assess its potential application as a marker for intact or functional cells and could be developed into a tool for monitoring the cellular state.

Following the track of the present work, IR microscopy could be translated into assessing the relationship between adhesion state and metabolism in the broader biological and biomedical research contexts. Within this perspective, our methodological work intends to set a reliable background for IR microscopy studies of cellular metabolism. By addressing its application to different morphological states of individual cells, we open the way to the characterization of more complex heterogenous cellular assemblies, with insight that would be inaccessible via bulk measurements.

## 4. Materials and Methods

### 4.1. HEK-cFXN Cell Line

The HEK-cFXN cell line used in these experiments has been described previously [16]. Briefly, the cell line was derived for other purposes from the immortalized commercial HEK line via knockout of the endogenous FXN gene, which expresses the protein frataxin, and replacing it with an exogenous, inducible, FXN gene to allow the fine-tuning of the frataxin levels. For the present work, the capability of this cell line to modulate frataxin expression was not exploited, and the conditions were tailored to result in physiological levels of the protein. We used these cells only as a convenient and already well-characterized model system.

HEK-cFXN cells were cultured in Dulbecco Modified Eagle Medium (DMEM), supplemented with 10% tetracycline-free foetal bovine serum (FBS; Clontech, Takara, Mountain View, CA), 10 mM sodium pyruvate, 2 mM L-glutamine, 2% (*v*/*v*) non-essential amino acids (NEAAs), 100 μg/mL hygromycin B, and 15 μg/mL blasticidin. Cells were cultured in an incubator at 37 °C with humidified air and 5% CO_2_. Unless differently stated, all reagents were acquired from ThermoFisher Scientific (Waltham, MA, USA). Sodium lactate was purchased from SIGMA-Aldrich (Sigma-Aldrich Chemie GmbH, Buchs, Switzerland).

### 4.2. Sample Preparation

HEK-cFXN cell samples were cultured in an incubator with a controlled atmosphere at 5% CO_2_ and only transferred into the sample holder for IR microscopy immediately before the measurement. FTIR spectra were acquired using either suspended or adherent HEK-cFXN cells. For suspended cell samples, HEK-cFXN cells were cultured overnight in 6-well plates and then prepared, immediately before the experiment, by detaching them with trypsin (Trypsin-EDTA 0.05%, Thermo Fisher Scientific, Waltham, MA) treatment for circa 30 s. Detached cells were resuspended in DMEM medium. The solution (10 µL) containing cells was transferred into the sample holder, namely, a liquid cell for IR microscopy (see Supplementary Information). For adherent cell samples, HEK-cFXN cells were cultured overnight on CaF_2_ windows, allowing them to adhere to the window surface. The day after, the CaF_2_ window with adhered cells was assembled with the other components of the sample holder. The empty volume created by the spacer between the two CaF_2_ windows (Crystran, Poole, UK) was filled with 10 µL DMEM medium. All samples were analysed by IR microscopy acquisition immediately after preparation in the sample holder. Once the cells were placed in it, no fresh medium was introduced, and the cells relied on the nutrients present in the volume of medium enclosed between the windows. The cell was sealed to prevent exchange of gas with the atmosphere. For the duration of the experiment, the sample holder was kept at 37 °C.

### 4.3. IR Microscopy Instrumentation

IR microscopy was performed at the MIRIAM beamline B22 of (Diamond Light Source, Didcot, UK) on a Hyperion 3000 IR microscope coupled to a Vertex 80 V interferometer (Bruker, Ettlingen, Germany), using a high sensitivity single element MCT (Mercury Cadmium Telluride) detector with size 50 microns and spectral cut off at ca. 650 cm^−1^. A broadband KBr beam splitter was used in the FTIR interferometer for covering the near/mid-IR spectral region (approx. 8000 to 400 cm^−1^). A Ge multilayer-coated filter was used in the optical path of the microscope to remove higher energy photons, thus defining the microscope illumination and spectral detection only in the mid-IR range (below circa 4000 cm^−1^). The IR beam was focused using a Schwarzschild condenser with 36× magnification and a numerical aperture 0.5. All measurements were performed in transmission mode, using a 36× objective identical to the condenser. The microscope was used in apertureless mode for all measurement by either synchrotron IR from beamline B22 or the thermal source (black body at circa 1000 K) inside the FTIR. The diameter and depth of focus of the diffraction-limited synchrotron light spot are a function of the IR wavelength [23]. At 6 µm, in the middle of the spectral range used in these experiments, the lateral resolution provided by a NA = 0.5 objective is circa 6 µm, smaller than the lateral dimension of both adherent and suspended cells. The corresponding depth of focus is circa 35 µm, a few times greater than the thickness of the 10 µm aqueous layer, implying that the expected lateral distribution of the beam is practically uniform across the sample. The light spot of the thermal source was not diffraction-limited under the conditions of operation, neither transversally nor longitudinally, and it is expected to be larger than the size of both adherent and suspended cells for the microscope optics used in these experiments. In agreement with these predictions, the integrated intensity of the microbeam over the full transmitted spectral range gave a Full-Width at Half Maximum (FWHM) of the detected spot of circa 15 µm at the sample plane (data not shown) by the diffraction limited synchrotron radiation source, and approximately 25 µm by the thermal source. Measurements were performed using either the synchrotron or the thermal source, depending on the required spatial distribution of the focal spot.

A custom sample holder for IR microscopy in aqueous environment (Suppl. Information and Suppl. Figure S1) was positioned on the automated motorized stage of the IR microscope to allow for sample inspection and measurement. The cells were enclosed in the holder and the visible light illumination of the microscope was used to inspect the sample with the help of a visible camera. Observation under visible light was used to assess sample movement.

### 4.4. Spectral Measurements and Analysis

We analyzed the data in terms of spectral variations, plotting the differential absorbance, rather than using the absolute spectra, because they highlight only the spectroscopic components changing over time (Note: Similar investigations of time-resolved changes by IR absorption spectroscopy reported in the literature often use the expression difference spectra, instead of spectral changes. The protocol used in these investigations often involves recording a background trace, followed by recording a sample trace in the sample location to obtain an absolute absorbance spectrum. A series of time-resolved absolute spectra is then recorded in the sample position, and the difference between the spectrum at time t and the spectrum at time 0 is used to calculate the difference spectrum at time t. This is analytically equivalent to the results of the measurement as performed in the present work, so that the terms difference spectra and spectral changes are equivalent). Individual cells were selected through their visible morphology as observed by the microscope visible light. In the case of adherent cells, we targeted the ones that appeared fully adherent to the surface with multiple filopodia and focused the beam on the central part of the cell. An initial spectral measurement was performed on the cell at time zero (t = 0), followed by repeated consecutive spectral measurements (single channel sample). The IR microbeam was maintained at the same sample location throughout the measurement for each cell. The single measurements were used to calculate spectral changes over time on the absorbance scale (see note). In contrast, absolute absorbance spectra were recorded by measuring the background spectrum on the medium and the sample spectrum on the cell itself.

FTIR scans were run in double-sided forward-backward acquisition mode at 4 cm^−1^ resolution using a nominal scan rate of 80 kHz (referred to the HeNe laser frequency of ca. 15,800 cm^−1^). A total of 256 scans were recorded and summed up for the background and for each measurement, corresponding to a total collection time for each spectrum of 32 s. Each cell was monitored for about 25 min after enclosure in the holder. The duration of the measurement was chosen to obtain an acceptable S/N ratio for lactate bands. Single spectra were obtained by Fourier transforming the interferogram after apodization with a Blackman–Harris 3-term function, using a zero-filling factor of 4 and a Mertz phase correction. Data were plotted as absorbance variations from the initial state over the time of the measurement. Spectra of single cells were recorded over time and analysed for the mid-IR spectral region. The thickness of the medium layer, 10 µm, led to high noise levels around 1640 cm^−1^ due to the water strong IR absorption (water bending mode). As the thickness of the CaF_2_ windows (twice 1 mm) reduced the usable spectral range to above 1000 cm^−1^, the IR spectral region appeared limited by noise at the extremes of the spectral interval, as well as in proximity of 1640 cm^−1^. Atmospheric changes (i.e., water vapour and gaseous carbon dioxide CO_2(g)_) were removed by the automated atmospheric correction algorithm in OPUS, which left minimal residual variations of atmospheric CO_2_ bands. Quantitative measurement of CO_2(aq)_ concentrations in liquid was instead performed after manual subtraction of CO_2(g)_ bands in gas phase.

Variations in the concentration of CO_2(aq)_ and HCO_3_^−^ were determined using the absorption coefficients by Falk and Miller (ε_(2343 cm^−1^)_ = 1.5 × 10^6^ cm^2^ mol^−1^ or 1.5 × 10^−4^ µm^−1^ mM^−1^ for CO_2_(aq) and ε_(1360 cm^−1^)_ = 0.4 × 10^6^ cm^2^ mol^−1^ or 4 × 10^−5^ µm^−1^ mM^−1^ ) [19].

### 4.5. Acidification

An estimate of final pH in the [CO_2(aq)_] and [HCO_3_^-^] buffer is provided from measured concentrations of using Equation (1):

pH = pK_a^−^_log ([CO_2(aq)_]/[HCO_3_^−^]) (1)

where K_a_ is the apparent acid dissociation constant for the serial equilibria involving hydration of aqueous CO_2(aq)_ and dissociation of carbonic acid to bicarbonate. In this work, we use pK_a_ = 6.04. [CO_2(aq)_] was determined from the measured increase in CO_2(aq)_ in the IR beam. [HCO_3_^−^] was estimated using an initial concentration of bicarbonate of 44 mM, as in the medium, and subtracting the amount of HCO_3_^−^_(aq)_ consumed in the IR beam. [CO_2(aq)_] was determined from the measured increase in CO_2(aq)_ in the IR beam under the assumption that the initial concentration of CO_2(aq)_ in the medium was negligible. Equation (1) is valid in the pH range where buffering capacity is dominated by CO_2(aq)_ and HCO_3_^−^_(aq)_, providing reliable values in the proximity of pH 6. Outside this range, multiple equilibria between other species should be considered. The equation also assumes that the local buffering equilibrium is established much faster than diffusion processes away from the probed volume [24].

### 4.6. Seahorse Experiments

One day (24 h) before the experiment, HEK-cFXN cells (adherent cell samples) were plated into Seahorse 96-well XF cell culture microplates (cat. no. 101085-004) at a density of 5 × 10^4^ cells per well. Cells were left overnight at 37 °C in humidified air supplemented with 5% CO_2_. The next day, the medium was changed to XF DMEM Medium (cat no. 103575-100) supplemented with 2 mM Glutamax (the pH of the medium was adjusted to 7.4 using 1 M NaOH). Cells were then incubated for 1 h at 37 °C in a CO_2_-free incubator. Suspension cells were prepared on the day of the experiment by incubating adherent HEK-cFXN cells in XF DMEM Medium (supplemented with Glutamax) for 45 min, detached with trypsin and, immediately before the measurement, deposited in the 96-well XF cell culture microplate at a density of 1 × 10^5^ cells per well. The 96-well XF cell culture microplate was pre-treated with Cell-Tak (22.4 μg/mL, Corning cat. no. 354240) to facilitate immobilisation of both suspension and adherent cells to the bottom of the microplate wells. The XF Glycolytic Stress Test (cat no. 103020-100) was carried out by loading the XF 96 Extracellular Flux assay sensor cartridge with glucose (final well concentration 10 mM), oligomycin (final well concentration 1.5 μM), and 2-DG (final well concentration 50 mM). Readings were normalized against the total protein content of each biological sample using a standard BCA assay (Thermo Scientific, cat. no. 23227). A positive variation corresponds to a decrease of pH. Statistical significance was tested using the unpaired t-test (** *p* < 0.01).

### 4.7. Data Reliability and Statistics

The experiments described in the present manuscript are the resultant of more than two months of experimental time distributed over six years during which we explored several different aspects and changed the parameters to test different conditions. In the present manuscript we describe only the results relevant to the methodological aspects of the experiment, i.e. the possibility to report differences in the turnover of suspended and adherent cells and the effect of synchrotron radiation on cells with one level of FXN expression. Data collected at different levels of FXN expression back up and increase the statistics of our results, even though not directly discussed in the manuscript. 

## Data Availability

Data are available from the authors upon reasonable request.

## Supplementary Materials

The following are available online at https://www.mdpi.com/article/10.3390/ijms221910742/s1
