# Supplementary material for: Label-Free, Real-Time Measurement of Metabolism of Adherent and Suspended Single Cells by In-Cell Fourier Transform Infrared Microspectroscopy
Source: Int J Mol Sci. 2021 Oct 4;22(19):10742. doi: 10.3390/ijms221910742 (PMC8509135; doi:10.3390/ijms221910742)
Supplement: Supplementary file 1 [file ijms-22-10742-s001.zip › ijms-1370434-supplementary.pdf]

Description of Sample Holder

The holder comprises a metal plate designed to integrate it with the microscope stage (**Figure S1**)[1]. A liquid sample is placed in the central bloc, which can be cooled by flowing a thermostatic fluid through an outer channel using a thermostatic bath circulator (Loop L100, from LAUDA-Brinkmann, Delran, NJ 08075, USA) (**Figure S1A**). The central bloc is designed as a classical sandwich holder for microliter samples, with two optical windows enclosing the liquid sample and separated by a gold-coated metal spacer. The thickness of the spacer is chosen to prevent sample damage and to provide the required optical path, typically of the order of micrometers or tens of micrometers. The material of the spacer can be varied depending on the required chemical and mechanical properties. The sandwich is pressed inside the metal chamber by rubber O-rings on both sides. The O-rings are compressed by tightening the screws of the upper plate when enclosing the cell (**Figure S1B,C**). A gap between the outer rim of the windows and the metal holder provides space to flow a fluid, such as fresh medium. If desired, the metal spacer can be cut to allow for diffusion between the circulating medium and the sample. For the experiments described in this work, the cells were located on the optical windows, covered with medium, and measured without flushing additional medium around the sample. A 10 μm gold coated metal spacer was used. The access ports to the medium channel where kept sealed to exclude the external atmosphere (**Figure S1B**).

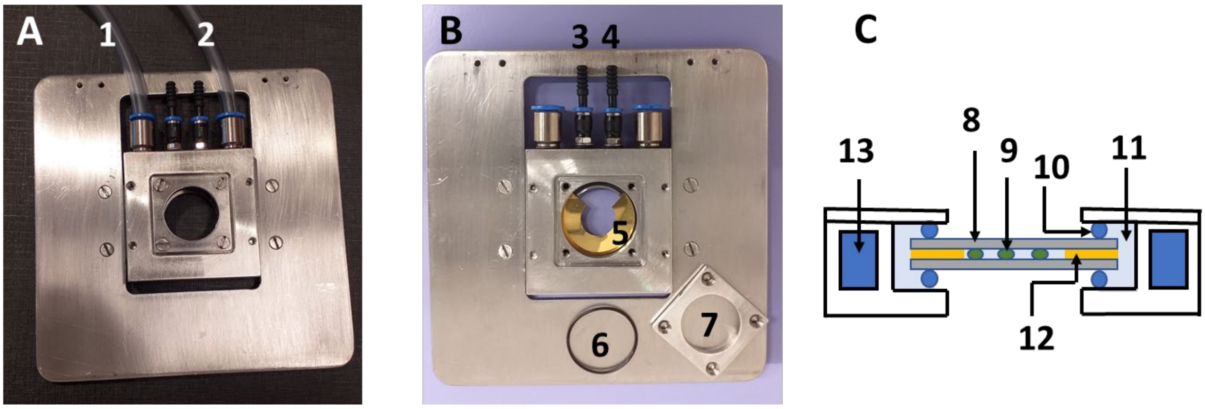

Figure S1. Sample Holder. (**A**) Closed holder without spacer and without windows. 1,2- inlet and outlet for thermostatic fluid. (**B**) Open holder with spacer and lower optical window. 3,4- inlet and outlet for medium flow. 5- spacer. 6- upper O-ring. 7- upper holding plate. (**C**) Scheme of cell environment. 8- optical windows. 9- cells and medium. 10- O-rings. 11- channel for medium. 12- spacer. 13- channel for thermostatic fluid flow.

Reference

1. Quaroni, L.; Zlateva, T.; Sarafimov, B.; Kreuzer, H.W.; Wehbe, K.; Hegg, E. L.; Cinque, G. Synchrotron Based Infrared Imaging and Spectroscopy via Focal Plane Array on Live Fibroblasts in D2O Enriched Medium. *Biophys. Chem.* **2014**, *189*, 40–48, https://doi.org/10.1016/j.bpc.2014.03.002.
